# Exploring the resilience and stability of a defined human gut microbiota consortium: An isothermal microcalorimetric study

**DOI:** 10.1002/mbo3.1430

**Published:** 2024-08-08

**Authors:** Anna Kattel, Valter Aro, Petri‐Jaan Lahtvee, Jekaterina Kazantseva, Arvi Jõers, Ranno Nahku, Isma Belouah

**Affiliations:** ^1^ Department of Chemistry and Biotechnology Tallinn University of Technology Tallinn Estonia; ^2^ Bioprocess Optimization Center of Food and Fermentation Technologies Tallinn Estonia; ^3^ Cell Biology University of Tartu, Institute of Technology Tartu Estonia

**Keywords:** consortium, gut microbiota, isothermal microcalorimetry, mucin, resilience, stability

## Abstract

The gut microbiota significantly contributes to human health and well‐being. The aim of this study was to evaluate the stability and resilience of a consortium composed of three next‐generation probiotics (NGPs) candidates originally found in the human gut. The growth patterns of *Akkermansia muciniphila*, *Bacteroides thetaiotaomicron*, and *Faecalibacterium prausnitzii* were studied both individually and consortium. The growth kinetics of Akkermansia muciniphila (*A. muciniphila*), Bacteroides thetaiotaomicron (*B. thetaiotaomicron*), and Faecalibacterium prausnitzii (*F. prausnitzii*) were characterized both individually and in consortium using isothermal microcalorimetry and 16S ribosomal RNA next‐generation sequencing. The consortium reached stability after three passages and demonstrated resilience to changes in its initial composition. The concentration of butyrate produced was nearly twice as high in the consortium compared to the monoculture of F. prausnitzii. The experimental conditions and methodologies used in this article are a solid foundation for developing further complex consortia.

## Graphical abstract

This research examined the stability and resilience of a consortium of human gut bacteria, namely *Akkermansia muciniphila*, *Bacteroides thetaiotaomicron*, and *Faecalibacterium prausnitzii*, in a nutrient‐rich medium using mucin as the carbon source. The study employed an isothermal microcalorimeter to monitor the growth of individual strains and their consortium. The results indicated a slight metabolic interaction between the strains, a stability and acute resilience of the consortium composition.

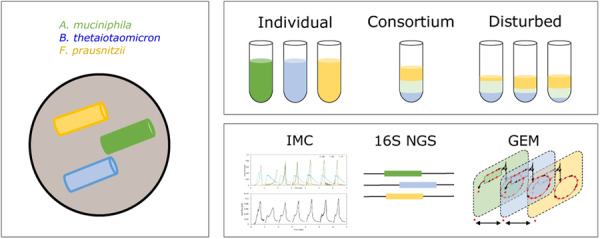

## 1 INTRODUCTION

The term “microbiota” has commonly been defined as the collection of microorganisms (bacteria, fungi, viruses, etc.) growing in a singular environment. For example, the gut microbiota is the collection of microorganisms colonizing and symbiotically existing within the gastrointestinal tract. Its composition plays an active role in the prevalence of various diseases (Type 2 diabetes mellitus, cancer, obesity, mental health) (Fan & Pedersen, 2021; Wang et al., 2017). The comparison of the gut microbiota from healthy and unhealthy individuals helped identify health‐beneficial phyla such as Firmicutes, Bacteroidetes, and Actinobacteria (Derrien & Veiga, 2017; Fan & Pedersen, 2021; Flint et al., 2012; Lopez‐Siles et al., 2017; Manor et al., 2020). In these phyla, some strains are currently considered potential next‐generation probiotics (NGPs) (Cani & Vos, 2017; Chang et al., 2019; Wrzosek et al., 2015) that could become an alternative or support to the faecal transplant (ClinicalTrials.gov identifier: NCT04208958, NCT03106844). To make this a reality, NGP strains should be characterized individually in vitro for specific attributes that would be preserved and improved in a complex functional NGP consortium. NGPs are looked upon for their capability to produce health‐beneficial molecules such as aromatic lactic acids, including indole‐lactate, and short‐chain fatty acids (SCFAs), including butyrate, propionate, acetate, and succinate (Gatto & Shliaha, 2013; Laursen et al., 2021; Rowland et al., 2018). SCFAs have anti‐inflammatory effects, innate immune functions, participate in intestinal homeostasis maintenance and epithelial barrier function, and serve as an energy source for the host epithelial cells and gut bacteria (Venegas et al., 2019). One of the most discussed SCFAs is butyrate (Coppola et al., 2021; Panebianco et al., 2022; Zhang et al., 2021). One butyrate‐producing bacteria is the *Faecalibacterium prausnitzii* within the *Faecalibacterium* genus (Zhu et al., 2021). *F. prausnitzii* preferentially consumes monosaccharides (glucose, rhamnose, fucose, galactose), disaccharides (cellobiose, lactose), amino sugars (N‐acetylglucosamine (N‐AcGam), N‐acetylgalactosamine (N‐AcGal)) and certain polysaccharides (pectin, starch) (Hu et al., 2022; Lopez‐Siles et al., 2012). Some of these carbohydrates (fucose, galactose, N‐AcGam, N‐AcGal) are the building blocks of mucin, a key component of the intestinal mucus that protects the gastrointestinal epithelium. The mucus layer serves as a functional barrier, protecting the epithelium from mechanical stresses and direct contact with pathogens. Additionally, it lubricates the passage of food and ensures the diffusion of small molecules (nutrients, ions, water and gases) (Paone & Cani, 2020). Mucin is a complex glycoprotein with a peptide backbone of repeated segments of proline‐threonine‐serine domains decorated with O‐glycans (galactose, N‐AcGam, N‐AcGal) chains (Ince et al., 2022) capped with sialic acid, sulfate, and fucose. To be used as substrates by *F. prausnitzii*, mucin has to be cleaved by gut microbiota members expressing specific carbohydrate‐activated enzymes (glycoside hydrolases, sialidases, sulfatases and esterases) (Raba & Luis, 2023). In their extensive genomic analysis of gut microbiota strains, Glover et al. (2022) confirmed the presence of mucin O‐glycan‐degrading hydrolases in *Akkermansia muciniphila* and *Bacteroides thetaiotaomicron* genome and their ability to grow using mucin as the sole carbon source in a chemically defined medium (ZMB1) (Glover et al., 2022; Kostopoulos et al., 2021). Additionally, *B. thetaiotaomicron* and *A. muciniphila* are established acetate‐producers that individually enhance the production of butyrate (by *F. prausnitzii* for example) in coculture (Chia et al., 2018; Kattel et al., 2023; Miner‐Williams et al., 2009; Paone & Cani, 2020; Wrzosek et al., 2013). However, there are no in vitro studies describing the stability and resilience of a consortium consisting of *A. muciniphila*, *B. thetaiotaomicron*, and *F. prausnitzii*.

*A. muciniphila*, *B. thetaiotaomicron*, and *F. prausnitzii* are regarded as obligate anaerobes. They are usually operated in anaerobic chambers which, besides the cost, are limited in space and require dexterity from the operator. These constraints intensify during continuous processes with extensive manual manipulations, like monitoring the growth. The most conventional method consists of using a spectrophotometer (600 nm) to track changes in the optical density (OD) over time. The sensitivity of this method reaches its limitations at high cell density and with solid, opaque, or non‐translucent media components, such as fibers (mucin, dietary fibers). Moreover, a spectrophotometer won't differentiate between metabolically active cells and nonviable ones. An alternative approach is the isothermal microcalorimetry (IMC). IMC is a non‐destructive, highly sensitive and high‐throughput method, with the possibility to monitor simultaneously up to 48 independent samples (Bayode et al., 2022; Braissant et al., 2015; Gaisford, 2016; Kabanova et al., 2012). The complexity of the samples is theoretically limitless (solid to liquid matrices, endothermic to exothermic processes). Each sample is placed in a glass ampoule and then hermetically sealed. The ampoule is then lowered into a heated sink maintained at the chosen temperature. A thermopile sensor, in contact with both the sample and heat sink (HS), detects the transfer of heat over time in both directions (sample‐to‐HS and HS‐to‐sample) (Braissant et al., 2015; Gaisford, 2016). The sensor measures continuously the heat flow released by metabolically active microorganisms. The heat flow is mathematically transformed into total heat, which is used to determine the specific growth rate. IMC becomes relevant in describing metabolic changes in systems where microbes interact with each other within a complex environment (mucin, dietary components).

To the best of our knowledge, this study is the first to monitor *A. muciniphila*, *B. thetaiotaomicron*, and *F. prausnitzii* consortium growth kinetics by IMC and 16S ribosomal RNA next‐generation sequencing (16S rRNA NGS), attesting to a robust and stable composition over time regardless of the initial consortium composition. Flux balance analysis (FBA) was tested to identify potential dependencies in their metabolisms in a complex environment. The authors are aware that the consortium used in the current study doesn't represent the huge complexity existing in the gut microbiome. Nonetheless, this study highlights the potential that exists in the continuous monitoring of NGP consortia for biotechnology advances and applications.

## 2 MATERIALS AND METHODS

### 2.1 Strains

*A. muciniphila* (DSM 22959), *B. thetaiotaomicron* (DSM 2079), and *F. prausnitzii* (DSM 17677) were ordered from DSMZ (German Collection of Microorganisms and Cell Cultures GmbH) and stored in 20% glycerol and PBS solution (pH 6.8) at −80°C. The *F. prausnitzii* genome was recently proposed as *Faecalibacterium duncaniae* sp. nov. (DSM 17677) (Sakamoto et al., 2022).

### 2.2 Preparation of yeast extract, casitone, fatty acids, and mucin culture medium (YCFAM)

The composition of the medium (if not stated otherwise) was as follows (per litre): 10 g casitone (Tryptone Plus, Sigma‐Aldrich), 2.5 g yeast extract (NuCel 545, Bio Springer), 4 g l‐cysteine HCl (Alfa Aesar), 1 mg resazurin sodium salt (Acros Organics), 2.93 g K_2_HPO_4_ (Sigma‐Aldrich), 4.65 g KH_2_PO_4_ (Sigma‐Aldrich), 0.9 g (NH_4_)_2_SO_4_ (Alfa Aesar), 0.9 g NaCl (Acros Organics), 0.28 g KOH (Fluka), 1 g NaHCO_3_ (Sigma‐Aldrich), 10 mg hemin (Sigma‐Aldrich), 10 µg biotin (Merck), 10 µg cobalamin (Fisher Bioreagents), 30 µg para‐aminobenzoic acid (Sigma‐Aldrich), 150 µg pyridoxamine (Carbosynth), 50 µg folate (Alfa Aesar), 0.09 g MgSO_4_·7H_2_O (Alfa Aesar), 0.09 g CaCl_2_·H_2_O (ApliChem). The medium was completed with a 1 mM concentration of acetaldehyde (Fluka) and SCFA, including acetate (Honeywell), propionate (Sigma‐Aldrich), valerate (Alfa Aesar), isovalerate (Acros Organics), isobutyrate (Acros Organics), formate (Fluka), lactate (Sigma‐Aldrich), l‐malate (Fluka), citrate (Sigma‐Aldrich), and fumarate (Fluka). Mucin from porcine stomach type III (Sigma‐Aldrich) was used as a substrate and included at 1.25 g L^−1^. SCFAs, vitamins, and hemin solution were filter‐sterilized and freshly added the day before the experiment. Mucin was sterilized at 115°C for 5 min before being added to the medium. The pH of the medium was adjusted to 7.1 using a 3 M NaOH solution. The final medium, called YCFAM, was kept overnight in an anaerobic chamber (COY box, Coy Laboratory Products Inc.) filled with 5% H_2_, 10% CO_2,_ and 85% N_2_ to remove traces of oxygen.

### 2.3 Culture growth conditions

Precultures were grown overnight in an anoxic YCFAM medium with 0.5% (w/v) glucose. Before inoculation, the cell culture stock was centrifuged at 14,000*g* for 1 min and resuspended in an anoxic YFCAM medium at a final OD (600 nm) of 0.62. All fermentations were performed under anaerobic conditions in microcalorimetry ampoules (2 mL working volume) containing YCFAM medium inoculated with bacterial culture (1% v/v). After inoculation, the ampoules were hermetically sealed and lowered into isothermal microcalorimeter channels (TAM III and TAM IV‐48, TA Instruments) set at 37°C. For each passage, 1% (v/v) of the culture (20 µL) from the prior batch was transferred into a new ampoule containing fresh YCFAM medium (1.98 mL). The growth profile was determined by continuously measuring the heat difference between the IMC control temperature (37°C) and the heat released by the bacterial metabolic activity. For the consortium fermentation, bacteria were prepared as described above. Heat curves from three biological replicates, from three independent IMC vials, were used to determine the mean and standard deviation of all the growth rates. Unless mentioned otherwise, the YCFAM medium was inoculated with 1% (v/v) of each bacterial culture. Each passage was performed as described above. At the end of the fermentation, for both individual strains and the consortium, the supernatant was collected after centrifugation (14,000*g*, 5 min, 4°C), culture composition was determined, and OD was measured.

### 2.4 Quantification of extracellular metabolites

Samples were centrifuged at 14,000*g* for 5 min at 4°C. The supernatant was transferred into a new tube and filtered through 0.2 µm syringe filters (Millipore Millex‐LG filters 13 mm Philic PTFE 0.2 µm non‐sterile SLLGH13NK; Millipore Corp.). The concentrations of acetate, citrate, butyrate, formate, isobutyrate, isovalerate, lactate, malate, propionate, and succinate were determined using high‐performance liquid chromatography (Waters 2695 HPLC system; Waters Corporation). The elution was done isocratically with 0.005 M H_2_SO_4_ solution at 0.6 mL min^−1^ using a 300 × 7.8 mm Aminex HPX‐87H column (Bio‐Rad), with precolumn 30 × 4.6 mm Micro‐Guard Cation H Refill Ca (Bio‐Rad). A 50/50 milliQ/MeOH (v/v) seal wash, 10/90 acetonitrile/milliQ (v/v) needle wash, autosampler temperature of 10°C, column temperature of 35°C, and injection volume of 20 µL were used. Refractive index (RI) and ultraviolet (UV) detectors (210 nm) (Waters Corporation) were used to detect and quantify metabolites using external standard curves. Standards were prepared by performing serial dilutions with milliQ water: 1x, 2x, 4x, 8x, 16x, 32x, 64x, and 128x.

### 2.5 DNA extraction and 16S rRNA NGS

The extraction of genomic DNA was done from the biomass pellet using a Sigma‐Aldrich GenElute Bacterial Genomic DNA kit and diluted to 10 ng µL^−1^. For library preparation, the V4 hypervariable region of the 16S rRNA gene was amplified with forward primer F515 5′‐GTGCCAGCMGCCGCGGTAA‐3′ and reverse primer R806 5′‐GGACTACHVGGGTWTCTAAT‐3′ (Caporaso et al., 2011) following a standard library preparation pipeline. The libraries were pooled at equimolar concentration and sequenced using the iSeq. 100 Sequencing System (Illumina) following a 2 × 150 cycles paired‐end sequencing protocol. Identification and quantification of the reads were done using BION‐META software (McDonald et al., 2016). The number of reads for each strain was normalized by the copy number of the 16S rRNA gene (3, 5, and 6 copies for *A. muciniphila*, *B. thetaiotaomicron*, and *F. prausnitzii*, respectively) (Stoddard et al., 2015).

### 2.6 Free amino acid quantification

The concentration of free amino acids was determined as described by Kivima et al. (2021) with a few modifications. Samples were thawed, vortexed and centrifuged (21,000*g*, 5 min, RT). Centrifuged samples were filtrated (Millipore Millex‐LG filters 13 mm Philic PTFE 0.2 µm non‐sterile SLLGH13NK; Millipore Corp.) and the supernatant was diluted 10 times with milliQ water. The free amino acids from the diluted supernatant were derivatized, separated with an AccQ‐Tag Ultra RP 1.7 µm (2.1 × 100 mm) column and quantified using a Waters UPLC‐PDA system (Waters). For derivatization, 6‐aminoquinolyl‐N‐hydroxysuccinimidyl carbamate (Synchem UG & Co) was used. The elution was carried out using a gradient of AccQTag Ultra eluents A and B (Waters). The data were processed with Empower 2 software (Waters).

### 2.7 Genome‐scale metabolic model (GEM)

#### 2.7.1 GEM source

The GEMs of *A. muciniphila*, *B. thetaiotaomicron*, and *F. prausnitzii* were collected from a public database published by Machado et al. (2018) (https://github.com/cdanielmachado/embl_gems). The GEMs were further curated using MEMOTE (metabolites formula, H^+^‐unbalanced reactions, duplicated reactions, unfeasible reactions, and futile cycles) until a consistency score higher than 95% was achieved (Lieven et al., 2020). For each strain, the biomass reaction was modified to integrate deoxyribonucleotides and ribonucleotides. In addition, the growth‐related ATP maintenance coefficient was adjusted. Amino acid transport reactions were added when missing. Mucin consumption was modeled by allowing the consumption of mucin‐derived monosaccharides (N‐AcGal, N‐AcGam, galactose, and fucose). With this approach, the energy expenditure associated with the cleavage of mucin was not accounted for. The consumption of mucin by *A. muciniphila* and *B. thetaiotaomicron* was supported by literature and validated experimentally (Degnan & Macfarlane, 1995; Derrien et al., 2004; Ottman et al., 2016). The exchange reactions associated with mucin‐derived monosaccharides were all set to zero in *F. Prausnitzii* GEM. Vitamins, ions (metals and salts), ribonucleotides, deoxyribonucleotides, and amino acids were not considered limited because of the presence of yeast extract in the medium. A summary of the GEMs can be found in Table S1. GEMs are provided in the supporting information section.

The GEMs were constrained using uptake and production rates calculated based on the difference in metabolite concentrations between the beginning and the end of fermentation, assuming most changes occurred before the stationary phase. Finally, the concentration difference was expressed per gDW and multiplied by the growth rate to obtain a rate (mmol (gDW h)^−1^). Data from the fifth to seventh passages were used to calculate an average and standard deviation (*SD*). The depletion of a metabolite from the medium was mimicked by constraining its exchange reaction to zero. A metabolite was considered growth‐limiting when its depletion led to a significant decrease in the predicted growth rate.

#### 2.7.2 GEM analysis

The pattern of intracellular fluxes was described using FBA and flux variance analysis (FVA) using the COBRA and RAVEN Toolbox (Becker et al., 2007) in MATLAB (The MathWorks Inc.), Gurobi solver (Gurobi Optimization Inc.), and by optimizing biomass production (growth rate). Both FBA and flux variability analysis (FVA; *n* = 5000 samples, 90% of maximal growth rate) were done using constraint‐based models. Experimental data obtained from the cultivations were used to constrain the model if not stated otherwise.

### 2.8 Data analysis

Quantification of extracellular metabolites was carried out with Empower software (Waters Corporation). Principal component analysis (PCA) was performed using R studio (v4.2.0) on centered and scaled data. For other analyzes, data were processed using Excel v20.04 (Microsoft Corporation). Figures were prepared using Inkscape (v1.2.1).

## 3 RESULTS AND DISCUSSION

### 3.1 Specific and reproductive growth pattern

In the current study, *A. muciniphila, B. thetaiotaomicron*, and *F. prausnitzii* were initially characterized in monoculture and then in the consortium, with the prior data sets used as references for the consortium.

**Figure 1:**
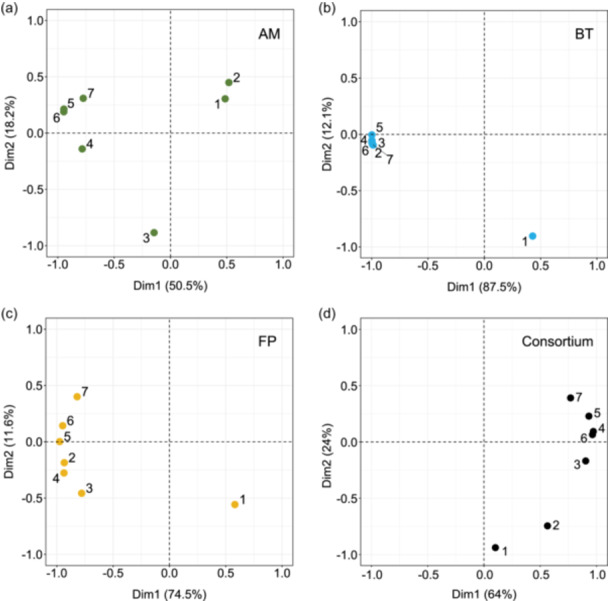
Three serial passages are required to reach consortium stability. Principal component analysis (PCA) was conducted using the heat flow monitored by IMC (*n* = 3) over seven successive passages (numbered from 1 to 7). Panels a–d correspond to *Akkermansia muciniphila* (green), *Bacteroides thetaiotaomicron* (blue), *Faecalibacterium prausnitzii* (yellow), and the consortium (black), respectively.

The characterization of the strains started by assessing their growth stability over several passages. A passage was defined by the transfer of 1% (v/v) of the preceding bacterial culture into fresh YCFAM medium, plus the fermentation time (usually 24 h). Passages were conducted daily and under strictly anaerobic conditions over 7 days. Numbers 1–7 are used in figures to refer to the passages, first to last. The growth kinetics of individual strains and the consortium were monitored using an isothermal microcalorimeter. The heat flow (W) data measured over the seven passages were summarized using a PCA (Figure 1). According to the PCA, the number of passages needed to reach stability (i.e. reproducibility in heat flow curves between passages), varied between individual strains and the consortium. The consortium required three passages to reach stability, while two passages were sufficient for *B. thetaiotaomicron* and *F. prausnitzii* monocultures (Figure 1b–d). At least four passages were needed before *A. muciniphila* monoculture showed stabilized growth kinetics (Figure 1a). From the fifth passage onward, the stability of the heat flow measurement was observed under all conditions. The heat flow from the fifth to seventh passages was used, after transformation into heat (J), to calculate growth rates. The existence of a linear relationship between heat and biomass was additionally confirmed before any growth rate calculations were made (Table S2).

The growth kinetics of *A. muciniphila* were characterized by a rapid increase of heat flow in the first 10 h after inoculation with a specific growth rate of 0.57 ± 0.02 h^−1^ (Figure S1A,B). This specific growth rate was slightly higher than the highest growth rate in basal medium reported by Noora et al. (2017) (0.41 ± 0.05 h^−1^). This difference could be attributed to the presence of yeast extract and casitone in our medium, as well as the higher concentration of mucin, which has been shown to trigger and modulate gene expression (Liu et al., 2021). The heat flow curves of *B. thetaiotaomicron* and *F. prausnitzii* showed multiple phases (Figure S1A,C,D). These phases were likely caused by metabolic switches triggered by the depletion of the most favorable substrate(s), thus reducing the growth rate at each new phase. For both *B. thetaiotaomicron* and *F. prausnitzii*, the highest growth rate occurred in the first few hours after inoculation (0.88 ± 0.06 h^−1^ and 1.00 ± 0.08 h^−1^, respectively) followed by a second growth phase (0.20 ± 0.01 h^−1^ and 0.21 ± 0.01 h^−1^, respectively) and a third phase (0.07 ± 0.002 h^−1^ and 0.09 ± 0.004 h^−1^, respectively). Prior genomic sequencing studies confirmed the presence and expression of mucin‐associated hydrolases and catabolizing pathways in *B. thetaiotaomicron* (Glover et al., 2022; Martens et al., 2011; Ravcheev et al., 2013). Moreover, Martens et al. (2011) already observed complicated polyphasic growth profiles with *B. thetaiotaomicron.* In mineral medium (ZBW1), *B. thetaiotaomicron* growth rate was 0.015 ± 0.002 h^−1^. This growth rate was seven times lower than the lowest measured in our YCFAM medium. This difference was most certainly explained by the consumption of other components (casitone, yeast extract) present in the YCFAM medium. In Lopez‐Siles et al. (2012) study, *F. prausnitzii* was unable to grow with mucin as a carbon source while in our YCFAM medium, its growth rate ranged from 0.09 ± 0.004 h^−1^ to 1.00 ± 0.08 h^‐1^. The comparison between our and Lopez‐Siles et al. (2012) medium showed that ours contained an additional six SCFAs, had a higher concentration of l‐cysteine and included phosphate buffers. Interestingly, growth rates obtained in the YCFAM medium were more consistent with those reported in the presence of mucin‐derived monosaccharides, such as N‐AcGam, glucosamine, or glucose (Duncan et al., 2002; Lopez‐Siles et al., 2012).

**Figure 2:**
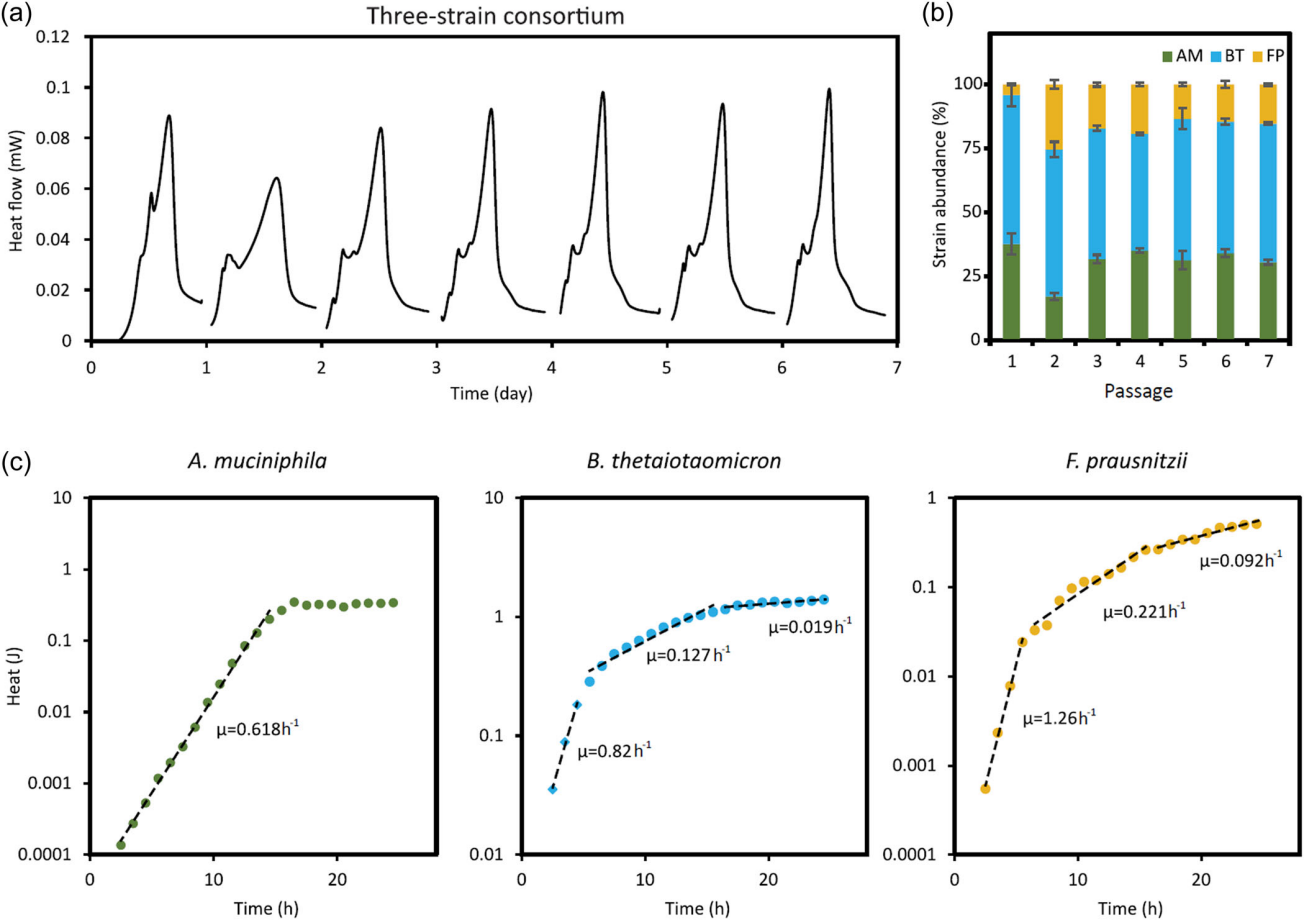
Robustness of consortium composition over serial passages. (a) The consortium growth was monitored in YCFAM over seven serial passages using the IMC. For each passage, the average and standard deviation of the heat flow (*n* = 3 replicates) are represented by a full line and ribbon, respectively. (b) At the end of passage cultivation, the consortium composition was determined using 16S rRNA sequencing. *A. muciniphila*, *B. thetaiotaomicron*, and *F. prausnitzii* are shown as green, blue, and yellow bars, respectively. (c) The growth rate (h^−1^) of the individual strains was calculated using the heat (J) released by the consortium and the abundance of each strain within the consortium. For 16S rRNA sequencing, samples were collected hourly over 24 h. rRNA, ribosomal RNA.

After individually characterizing each strain, *A. muciniphila*, *B. thetaiotaomicron*, and *F. prausnitzii* were co‐inoculated in the YCFAM medium, starting with the same initial cell density. The three‐strain consortium growth was monitored by IMC over seven passages and compared to the individual strains (Figure S1A). In accordance with PCA (Figure 1d), the robustness of the consortium composition improved with the number of passages. From the fourth passage, the consortium heat flow curves were comparable to the combination of the curves from individual strains (Figure S1A). To confirm that the heat flow released by the consortium originated from the metabolic activities of all three strains, their presence was confirmed by 16S rRNA sequencing (Figure 2b). *B. thetaiotaomicron*, *A. muciniphila*, and *F. prausnitzii* represented 52 ± 2%, 34 ± 2%, and 13 ± 1% of the final consortium, respectively (Table S3).

As a further step in consortium characterization, the growth rate of individual strains within the consortium was determined. The abundance of each strain within the consortium was quantified hourly over 24 h using 16S rRNA amplicon sequencing. These abundances were used to reconstruct the growth curve of individual strains from the total heat generated by the consortium. Regardless of the strain, the growth rates calculated from the reconstructed curves resembled those obtained with monoculture cultivations (above paragraph and Figure 2c). In this condition, the results suggested that *B. thetaiotaomicron*, *A. muciniphila*, and *F. prausnitzii* stably coexist in the YCFAM medium.

### 3.2 Evaluation of consortium resilience through growth kinetics and 16S rRNA NGS

In their work, Allison and Martiny (2008) defined resilience as the ability of a community to quickly recover from a disturbance whether by growth, physiological, or genetic adaptation. Recent works suggested that pH, medium composition, taxonomic group, and species initial abundance influence community stability over serial passages (Goldford et al., 2018; Segura Munoz et al., 2022; Zegeye et al., 2019).

**Figure 3:**
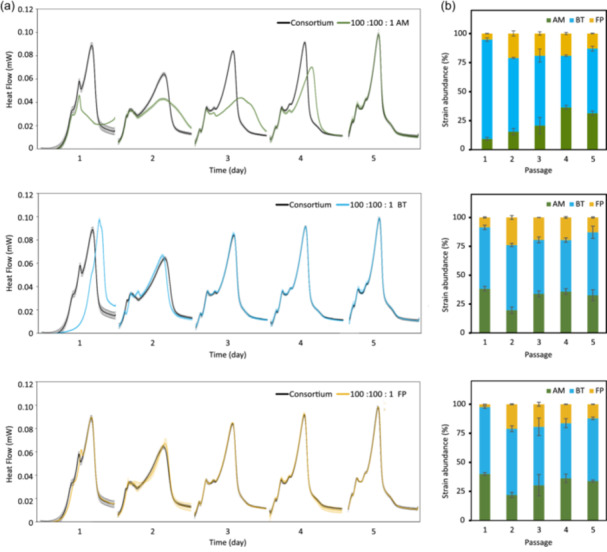
Resilience of the consortium community. (a) The resilience of the consortium was tested across three combinations. In each combination, *A. muciniphila*, *B. thetaiotaomicron*, or *F. prausnitzii* strain was inoculated at a 100‐fold lower concentration compared to the two others (top to down, respectively). The full line and ribbon represent the average and standard deviation of the heat flow monitored in triplicates. The resilience was reached when the original heat flow (black, Figure 2a) was recovered. (b) The abundance of *A. muciniphila* (green), *B. thetaiotaomicron* (blue), and *F. prausnitzii* (yellow) was determined using 16S rRNA sequencing at the end of every passage (*n* = 3). rRNA, ribosomal RNA.

To assess the resilience of our consortium, the effect of species’ initial abundance was tested across three combinations (Figure 3). In each combination, one strain was inoculated at a concentration a hundred times lower than the other two strains. The composition of the consortia was evaluated at the end of each passage. Resilience was achieved when the growth kinetics and composition of a combination closely resembled the reference (Figure 3a).

The growth kinetics were significantly altered from the reference consortium when the initial concentration of BT and AM were lowered (Figure 3a, first two panels). A similar growth kinetics to the reference was retrieved after the second and fifth passages, respectively. Similarly to *B. thetaiotaomicron*, two passages were also sufficient for *F. prausnitzii* (Figure 3a, third panel). In coherence with the heat flow, a return to the former consortium composition was also obtained by 16S rRNA sequencing at the end of the fifth passage (Figure 3b [passage 5] and Figure 2b [passages 5–7]). The composition of the consortia from passages 1–5 is reported in Table S4. This return could not be achieved in the event of drastic competition for growth‐dependent substrate(s).

### 3.3 A slight interaction is suggested by changes in organic acid concentrations

**Figure 4:**
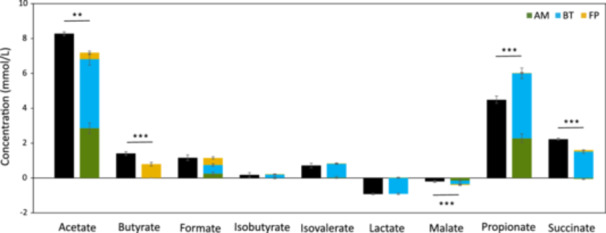
External metabolite production in monocultures and the consortium. Organic acids (mmol/L) were quantified at the end of the fifth, sixth, and seventh passages. Green, blue, and yellow bars represent individual cultures—*A. muciniphila*, *B. thetaiotaomicron*, and *F. prausnitzii* respectively, and black bars represent the consortium. The concentrations from individual strains were stacked up to visualize the expected concentrations in the consortium in the absence of interactions between the species. Statistical significance between the consortium and the sum of individual cultures is calculated with a Student's *t*‐test (*p < 0.05, **p < 0.01, ***p < 0.001).

Although the three strains proved able to thrive in the consortium, some metabolic changes may have been necessary to maintain the stability. To evaluate this hypothesis, the production and consumption of extracellular organic acids were quantified at the end of monoculture and consortium cultivations (Table S5). As reported in the literature, *F. prausnitzii* was characterized by the secretion of butyrate and formate (Figure 4) (D'hoe et al., 2018; Lopez‐Siles et al., 2012; Wrzosek et al., 2013). However, the consumption of acetate by *F. prausnitzii* was not confirmed in our study. *B. thetaiotaomicron* and *A. muciniphila* showed similar excretion profiles, with significant production of acetate and propionate, and consumption of malate (Das et al., 2018; Noora et al., 2017; van der Ark et al., 2018). The secretion of both isovaleric and isobutyrate and the consumption of lactate were measured only with *B. thetaiotaomicron*. The consortium had a significantly higher concentration of acetate, butyrate, and succinate compared to the combination of concentrations produced by individual strains (Figure 4; Table S5). The higher production of butyrate in the consortium was not correlated with the expected decrease in acetate concentration suggested by Wrzosek et al. (2013). However, at the end of the consortium fermentation, the concentration of acetate was eight times higher compared to *F. prausnitzii* monoculture in YCFAM, which could have enhanced butyrate production by *F. prausnitzii* (Figure 4).

### 3.4 Free amino acid consumption in the consortium is mainly influenced by *B. thetaiotaomicron*

In addition to organic acids, the concentrations of 20 free amino acids were assessed at the end of the fifth, sixth, and seventh passages.

The difference in secretion and consumption of free amino acids between individual strains and consortium was examined using PCA and including the initial medium composition as a reference. The first and second components (PCs) explained 99.9% of the variability (Figure S2). The consortium and *B. thetaiotaomicron* were separated from *F. prausnitzii*, *A. muciniphila* and the medium samples in PC2. This result suggested that in the consortium the free amino acids consumption in the consortium was mainly driven by *B. thetaiotaomicron*. At the end of *A. muciniphila* and *F. prausnitzii* cultivations, the concentration of the free amino acids was comparable to the reference, suggesting either a negligible or no consumption of any free amino acids (Table S6)*.* This observation differed greatly from the net consumption and excretion of 10 amino acids by *F. prausnitzii* reported by Heinken et al. (2014). In addition, our data did not confirm threonine consumption by *A. muciniphila* as predicted by Noora et al. (2017) and reported by van der Ark et al. (2018) (Table S6). At the end of *B. thetaiotaomicron* cultivation, over half of the measured free amino acids had increased in the medium (Table S6). Phenylalanine and tryptophan concentrations remained unchanged. In comparison to the reference, the abundances of proline and glutamine were higher. Furthermore, aspartate and asparagine were depleted. Data reported by Catlett et al. (2020) suggested that *B. thetaiotaomicron* uses l‐asparagine hydrolase (EC 3.5.1.1; BT_0526, BT_2404, BT2757) to convert asparagine to aspartate. Aspartate can then enter the TCA cycle as oxaloacetate (EC 2.6.1.1; BT_2415), resulting in the generation of ATP and cofactor (NADPH). From the overflow in the TCA cycle, the succinate that is produced by succinyl‐CoA ligase (EC 6.2.1.5; BT_0788, BT_0787) is either secreted and/or mobilized in the respiratory chain to generate the proton gradient required for ATP production (Catlett et al., 2020). In addition to asparagine and aspartate, serine was also consumed and can be converted into pyruvate (EC 4.3.1.17; BT_4678).

### 3.5 Metabolic modeling suggests distinct growth‐limiting substrates for individual strains

**Figure 5:**
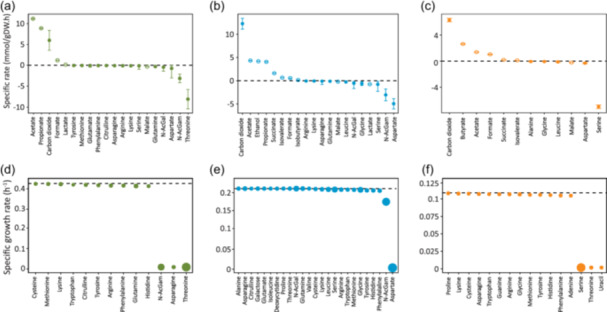
Predicted metabolic consumption and production rates for *A. muciniphila*, *B. thetaiotaomicron*, and *F. prausnitzii*. (a–c) Production and consumption rates predicted by FBA for *A. muciniphila* (a), *B. thetaiotaomicron* (b), and *F. prausnitzii* (c). Open circles show fluxes predicted by FBA, while plain circles represent fluxes that were calculated from experimental data and used to constrain respective models. Production and consumption are represented by positive and negative rates, respectively. Fluxes with absolute values lower than 0.1 were considered negligible and omitted. Specific rates were averaged, and standard deviations were calculated after flux variability analysis (*n* = 5000 samples). (d–f) Effect of substrate depletion on specific growth rates of *A. muciniphila*, *B. thetaiotaomicron*, and *F. prausnitzii*. To mimic the depletion of a substrate from the medium, the lower bound of its exchange reaction was set to zero. The dashed line represents the optimal growth rate without limitation. The size of the dots is relative to the priorly predicted uptake rates. FBA, flux balance analysis.

In this study, FBA was used to identify strain‐specific and shared carbon sources. It was assumed that all strains could uptake and catabolize amino acids and that *F. prausnitzii* was the only strain unable to cleave, and thus consume, mucin‐derived monosaccharides. *A. muciniphila*, *B. thetaiotaomicron*, and *F. prausnitzii* genome‐scale metabolic models (GEMs) consisted of 1308, 1491, and 1137 metabolic reactions, respectively (Table S1). GEMs were constrained using experimental rates (Table S7, Section 2.7.1). The analysis looked for the flux distributions for which the production of biomass, i.e*.*, specific growth rate, was the most optimized (Tables S8–S10). It is important to mention that an average experimental growth rate was calculated and used to constrain GEMs for *B. thetaiotaomicron* and *F. prausnitzii.* Growth of *A. muciniphila* was predicted to be mainly sustained by threonine and N‐AcGam consumption (Figure 5a). Threonine was predicted to enter the TCA cycle as succinyl‐CoA to generate ATP. In addition, threonine was converted to glycine and serine. These predictions were not confirmed experimentally (Table S6).

*B. thetaiotaomicron* was predicted to consume preferentially aspartate and N‐AcGam (Figure 5b). As described in Section 3.3 for *B. thetaiotaomicron*, aspartate entered the TCA cycle to generate ATP, leading to the excretion of succinate and propionate. For both *A. muciniphila* and *B. thetaiotaomicron*, N‐AcGam entered glycolysis as fructose 6‐phosphate in three metabolic reactions. The energy (ATP) required to grow was generated by glycolytic enzymes (pyruvate kinase PYK, phosphoglycerate kinase PGK) and TCA enzyme (succinyl‐CoA synthetase SUCOAS) (Tables S8–S10). In *A. muciniphila* metabolism, ATP was actively used by formate tetrahydrofolate ligase (FTHFLi) and phosphofructokinase (PFK). For *B. thetaiotaomicron*, the prediction showed that ATP turnover was highest for N‐acetylglucosamine kinase (ACGAMK) and glucose‐1‐phosphate adenylyl transferase (GLGC). In the absence of evidence suggesting that *F. prausnitzii* could cleave mucin and thus consume any of the monosaccharides, FBA predicted serine to be the main carbon source for *F. prausnitzii* (Figure 5c). Serine was predicted to enter central carbon metabolism after being converted by l‐serine ammonia‐lyase (SERD_L) to pyruvate. This result was consistent with the data reported in Auger et al. (2022), showing a higher expression of serine/threonine transporter (SstT) at an early stage of fermentation. However, the depletion or consumption of serine by *F. prausnitzii* was not experimentally confirmed (Table S6). Acetate kinase reaction (ACKr) was predicted as an almost unique ATP source for *F. prausnitzii*, with one ATP produced per acetate catabolized. The limited use of the glycolysis pathway to generate ATP was due to the absence of mucin‐derived monosaccharides consumption.

The following step aimed to determine which of the predicted carbon sources were essential to sustain growth and which were potentially shared by species (Table S11). The depletion of a substrate from the medium was mimicked by setting the consumption rate to zero while optimizing biomass production. In the case of *A. muciniphila*, the depletion of N‐AcGam, asparagine, or threonine entirely stopped the growth, while the other substrates had only a slight effect on the predicted growth rate (Figure 5d). These results were coherent with a previous study where N‐AcGam and l‐threonine were essential for the growth of *A. muciniphila* in a minimal medium (van der Ark et al., 2018). Although asparagine was not shown as an essential nutrient, its consumption was confirmed in a BHI medium supplemented with mucin (Liu et al., 2021). *B. thetaiotaomicron*, the most robust strain, had the widest range of substrates and was slightly affected by the depletion of most of its carbon sources (Figure 5e). Only the depletion of aspartate or N‐AcGam had a significant effect on its growth rate. For *F. prausnitzii*, serine, threonine, and uracil turned out to be essential for its growth in this specific condition (Figure 5f). Interestingly, the omission of serine, threonine, and uracil in the defined medium used by Heinken et al., (2014) still allowed the growth of *F. prausnitzii*. Nonetheless, the growth in this medium was described by the authors as poor and inconsistent (lower than 0.13 h^−1^). Although predicting fluxes using a complex medium is controversial, our results suggested that threonine and N‐AcGam were the common substrates that strains could compete for within the consortium via which they could alter each other's growth.

## 4 CONCLUSION

This study demonstrates the stability, reproducibility, and resilience of a potential NGP consortium. Our results showed that a stable and reproducible composition was reached within several passages and could be maintained thereafter. Despite significant disturbances in its original composition, the resilience of the consortium could be demonstrated by recovering its expected growth kinetics and composition. The production of acetate and propionate by *A. muciniphila* and *B. thetaiotaomicron* was confirmed. The secretion of butyrate by *F. prausnitzii* was higher when grown in a consortium, likely due to the increased concentration of acetate available in the medium. In silico analysis predicted that all three species preferred mucin‐derived saccharides (N‐AcGAM and N‐AcGal) as substrates. This competition was most likely compensated in nutrient‐rich YCFAM medium to allow all strains to thrive in consortium. To the best of our knowledge, this is the first time that the resilience of these NGP candidates has been dynamically monitored and confirmed by 16S rRNA NGS analysis. Continuous monitoring throughout fermentation was made possible using IMC confirming the high potential of the device, even in an opaque environment. IMC represents an alternative methodology that responds to the demand for continuous monitoring of bacterial growth kinetics (Lebas et al., 2020). These methodologies could be further used for more complex consortia with additional environmental disturbances (pH, temperature, nutrients).

## AUTHOR CONTRIBUTIONS

**Anna Kattel**: Data curation; methodology; writing—review and editing (equal). **Valter Aro**: Data curation; methodology; writing—original draft (equal). **Petri‐Jaan Lahtvee**: Funding acquisition; investigation; supervision (equal). **Jekaterina Kazantseva**: Writing—review and editing (equal). **Arvi Jõers**: Conceptualization; funding acquisition; investigation; project administration; supervision (equal). **Ranno Nahku**: Conceptualization; data curation; funding acquisition; methodology; project administration; resources; writing—review and editing; supervision (equal). **Isma Belouah**: Data curation; formal analysis; methodology; visualization; writing—review and editing; writing—original draft (equal).

## CONFLICT OF INTEREST STATEMENT

None declared.

## ETHICS STATEMENT

None required.

## Supporting information

Figure S1 **Growth kinetics of the individual strains and the consortium in reference condition. A**, Heat flow (W) released by individual strains and consortium. Growth was monitored in YCFAM over seven serial passages using the IMC. For each passage, the average and standard deviation of the heat flow (n = 3 replicates) are represented by a full line and ribbon, respectively. Green, blue, yellow, and black lines represent *A. muciniphila*, *B. thetaiotaomicron*, *F. prausnitzii*, and the consortium, respectively. **B‐D**, For each strain, specific growth rate(s) were determined at each phase using heat (J) from the fifth, sixth and seventh passage. The full line and ribbon represent the average heat and standard deviation of the fifth, sixth and seventh passage (n = 3 replicates).

Figure S2 **The free amino acid profiles of** ***B. thetaiotaomicron*** **are similar to the consortium.** Principal component analysis (PCA) was conducted using the concentrations (mmol/L) of twenty free amino acids. Concentrations were quantified in triplicates at the end of the fifth, sixth, and seventh passages. In addition to individual strains and the consortium, a reference (blank medium sample) was included in the analysis. Green, blue, yellow, black and grey markers represent *A. muciniphila*, *B. thetaiotaomicron*, *F. prausnitzii*, the consortium and the reference, respectively.

Supporting information.

## Data Availability

The data that support the findings of this study are openly available in FastMicro at https://github.com/Tftak-IB/FastMicro.git. Additional supporting information, including supplemental tables, figures and GEMs, can be found on GitHub: https://github.com/Tftak-nIB/FastMicro.git.
