# Supplementary figures and images for: Exploring the resilience and stability of a defined human gut microbiota consortium: An isothermal microcalorimetric study
Source: Microbiologyopen. 2024 Aug 8;13(4):e1430. doi: 10.1002/mbo3.1430 (PMC11307317; doi:10.1002/mbo3.1430)

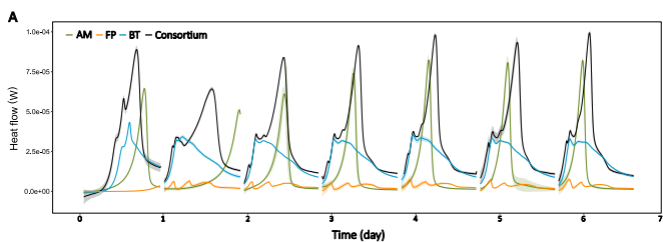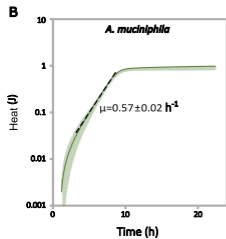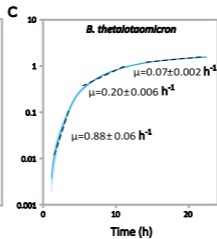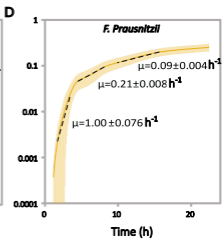

Supplement: Supplementary file 1 — Figure S1 Growth kinetics of the individual strains and the consortium in reference condition. A, Heat flow (W) released by individual strains and consortium. Growth was monitored in YCFAM over seven serial passages using the IMC. For each passage, the average and standard deviation of the heat flow (n = 3 replicates) are represented by a full line and ribbon, respectively. Green, blue, yellow, and black lines represent A. muciniphila, B. thetaiotaomicron, F. prausnitzii, and the consortium, respectively. B‐D, For each strain, specific growth rate(s) were determined at each phase using heat (J) from the fifth, sixth and seventh passage. The full line and ribbon represent the average heat and standard deviation of the fifth, sixth and seventh passage (n = 3 replicates). [file MBO3-13-e1430-s003.pdf]

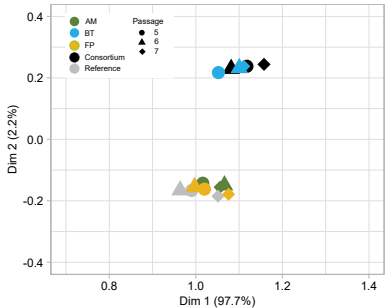

Supplement: Supplementary file 2 — Figure S2 The free amino acid profiles of B. thetaiotaomicron are similar to the consortium. Principal component analysis (PCA) was conducted using the concentrations (mmol/L) of twenty free amino acids. Concentrations were quantified in triplicates at the end of the fifth, sixth, and seventh passages. In addition to individual strains and the consortium, a reference (blank medium sample) was included in the analysis. Green, blue, yellow, black and grey markers represent A. muciniphila, B. thetaiotaomicron, F. prausnitzii, the consortium and the reference, respectively. [file MBO3-13-e1430-s001.pdf]
